# Supplementary material for: Risk assessment and mitigation evaluation of future yellow fever outbreaks under different climate scenarios: Insight from a case study of Brazil
Source: PLoS Negl Trop Dis. 2025 Oct 16;19(10):e0013448. doi: 10.1371/journal.pntd.0013448 (PMC12543279; doi:10.1371/journal.pntd.0013448)
Supplement: S1 Appendix — (PDF) [file pntd.0013448.s001.pdf]

1 Mathematical Model and Basic Reproduction Number Derivation

The corresponding ODE system is given by

$$
\text{Humans}\quad
\begin{cases}
\dfrac{\mathrm{d}S}{\mathrm{d}t} &= -\beta_1 SZ - vS,\\[2ex]
\dfrac{\mathrm{d}V}{\mathrm{d}t} &= vS - (1-\eta)\beta_1 VZ,\\[2ex]
\dfrac{\mathrm{d}E}{\mathrm{d}t} &= \beta_1 SZ + (1-\eta)\beta_1 VZ - \lambda_1 E,\\[2ex]
\dfrac{\mathrm{d}I}{\mathrm{d}t} &= \phi\lambda_1 E - (\gamma+\omega)I,\\[2ex]
\dfrac{\mathrm{d}A}{\mathrm{d}t} &= (1-\phi)\lambda_1 E - \gamma A,\\[2ex]
\dfrac{\mathrm{d}R}{\mathrm{d}t} &= \gamma(I+A),\\[2ex]
\dfrac{\mathrm{d}D}{\mathrm{d}t} &= \omega I,
\end{cases}
\tag{Eq 1}
$$

$$
\text{Mosquitoes}\quad
\begin{cases}
\dfrac{\mathrm{d}X}{\mathrm{d}t} &= \delta_1(T)\delta_2(\zeta)Me^{-\sigma M} - \beta_2 X(I+A) - \mu(T)X,\\[2ex]
\dfrac{\mathrm{d}Y}{\mathrm{d}t} &= \beta_2 X(I+A) - (\lambda_2(T)+\mu(T))Y,\\[2ex]
\dfrac{\mathrm{d}Z}{\mathrm{d}t} &= \lambda_2(T)Y - \mu(T)Z,
\end{cases}
\tag{Eq 2}
$$

where $M(t) := X(t) + Y(t) + Z(t)$ gives the total mosquito population at time t.

The temperature and rainfall-dependent parameters are given by the following functions:

$$\delta_1(T)\delta_2(\zeta) := (0.03T - 0.32)\cdot\frac{(1+s)e^{-r(\zeta-R_*)^2}}{s+e^{-r(\zeta-R_*)^2}}, \tag{Eq 3}$$

$$\mu(T) := \frac{7}{\mu_c(T-12.7137)(38.0481-T)}, \tag{Eq 4}$$

$$\lambda_2(T) := 0.0007T(T-17.67)\sqrt{42.11-T}. \tag{Eq 5}$$

The numerical values of the above parameters are $s = 8.4, r = 0.0035$, and $R_* = 29$. In our work, we make the assumption that the impact of rainfall on mosquito development is intrinsic to

the mosquitoes and independent of the geographical location. Hence, we assume that the parameter estimates in Abdelrazec et al.[22], which come from modelling done in Ontario, Canada, may also be used for the modelling of mosquito dynamics in Brazil. The death rate and extrinsic incubation functions take the form of the estimates in Gaythorpe et al.[22]. We note that the parameter μ_c in Gaythorpe et al.[22], which appears in the mosquito mortality rate, was re-estimated via the nonlinear least squares method, giving $\mu_c = 0.18$. The difference in this parameter value can likely be attributed to various factors: different mosquito genus being considered in Brazil vs the regions in Gaythorpe et al.[22] and differences in mortality rates in these different regions under the same temperature regimes.

To address the physical validity of the model, a proof of the well-posedness of model (Eq 1) – (Eq 2) is provided in Appendix S3.

In Section 2, we presented the basic human infection reproduction number, $\mathcal{R}_H$, which is a function of several key parameters, including the average temperature value since μ and λ_2 depend on temperature.

$$\mathcal{R}_{\mathcal{H}} = \frac{\beta_1 S_0 \lambda_2}{\mu(\mu + \lambda_2)}.$$

$\beta_1 S_0$ represents the number of successful disease-transmitting contacts per week that an infected mosquito would make with a fully susceptible population. The expression $\frac{\lambda_2}{\mu(\mu+\lambda_2)}$ represents the average infectious period of a mosquito, including both the exposed and infectious stages.
