# Supplementary material for: Risk assessment and mitigation evaluation of future yellow fever outbreaks under different climate scenarios: Insight from a case study of Brazil
Source: PLoS Negl Trop Dis. 2025 Oct 16;19(10):e0013448. doi: 10.1371/journal.pntd.0013448 (PMC12543279; doi:10.1371/journal.pntd.0013448)
Supplement: S2 Appendix — (PDF) [file pntd.0013448.s002.pdf]

2 Supplementary Figures

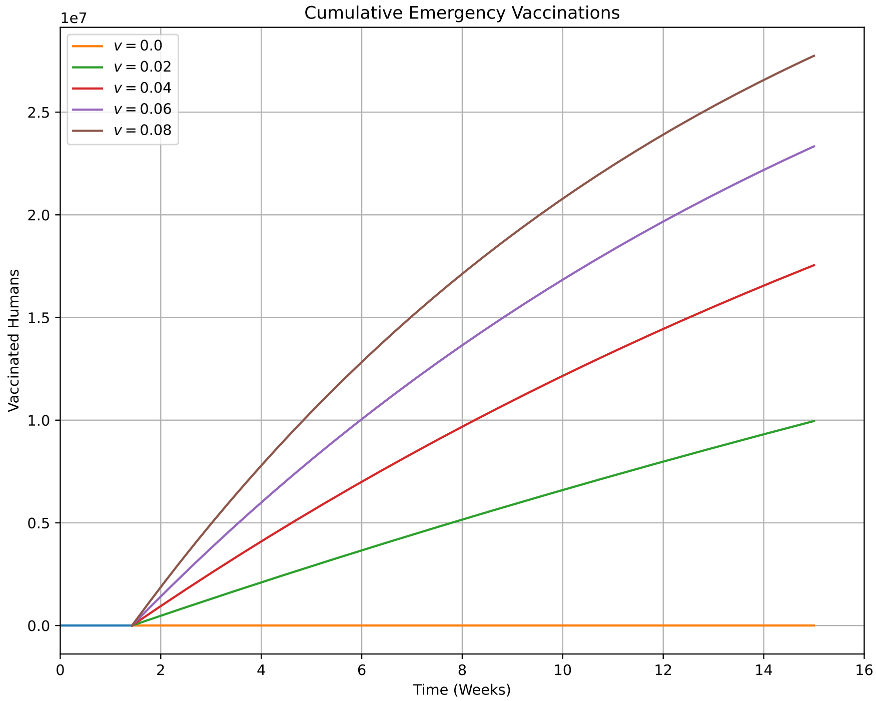

Figure A The impact of different vaccination rates (v) on the cumulative emergency vaccination doses required over the course of 15 weeks.

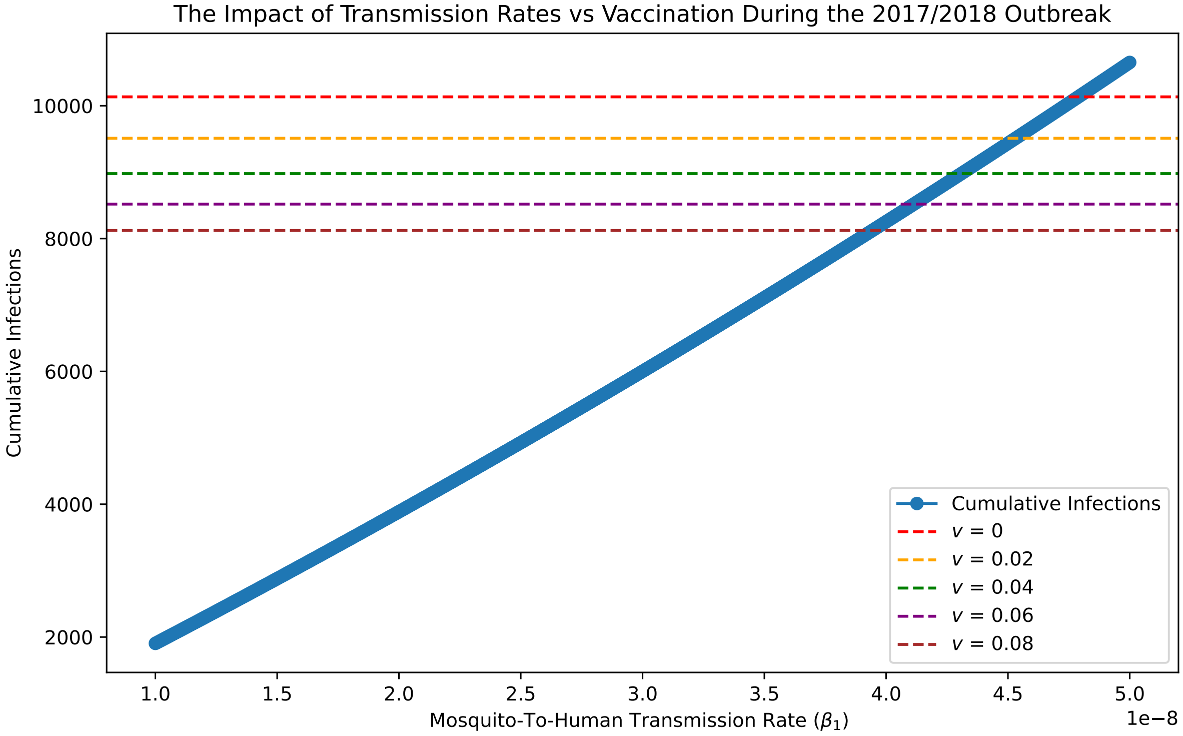

Figure B The equivalence between reducing the mosquito-to-human transmission rate and administering additional emergency vaccines under the conditions of the 2017/2018 outbreak.
