# Supplementary material for: Risk assessment and mitigation evaluation of future yellow fever outbreaks under different climate scenarios: Insight from a case study of Brazil
Source: PLoS Negl Trop Dis. 2025 Oct 16;19(10):e0013448. doi: 10.1371/journal.pntd.0013448 (PMC12543279; doi:10.1371/journal.pntd.0013448)
Supplement: S3 Appendix — (PDF) [file pntd.0013448.s003.pdf]

3 Well-Posedness

In order to assess biological validity, we need to address the well-posedness of system (Eq 2)–(Eq 3) from S1, in the sense of non-negativity and boundedness of solutions given non-negative initial conditions. We note that existence and uniqueness of solutions follows from the classical fundamental theory for first-order ODE systems.

Assume we have non-negative initial conditions. Consider the first equation of system S1 (Eq 2). It follows that the solution satisfies

$$S(t) = S(0)\exp\left(-\int_0^t \beta_1 Z(\xi) + v\mathrm{d}\xi\right).$$

Hence, since $S(0) \geq 0$, it follows that $S(t) \geq 0$ for all $t \geq 0$. In fact, the inequality can be made strict if the initial susceptible population is positive. Similarly, it follows from the variation of constants formula and the equation for the V compartment that the solution must satisfy

$$V(t) = V(0)\exp\left[-(1-\eta)\beta_1\int_0^t Z(\psi)\mathrm{d}\psi\right] + v\int_0^t\left[S(\xi)\exp\left(-(1-\eta)\beta_1\int_\xi^t Z(\psi)\mathrm{d}\psi\right)\right]\mathrm{d}\xi.$$

Since we have $S(t) \geq 0$ for all $t \geq 0$ and $V(0) \geq 0$, it follows that $V(t) \geq 0$ for all $t \geq 0$.

To establish non-negativity of the remaining components of the solution, we may apply Theorem 2.1 in Chapter 5 of Smith[40]. In particular, we assume to the contrary that at some time $t = t^*$, at one of the components of the solution becomes negative. By continuity, these solution must first cross 0. Assume that $E(t)$ is the first component to become negative at time $t = t^*$, i.e., $E(t^*) = 0$. Plugging $E = 0$ into system S1 (Eq 2) – (Eq 3), the equation for E yields

$$\left.\frac{\mathrm{d}E}{\mathrm{d}t}\right|_{t=t^*} = \beta_1\left[S(t^*) + (1-\eta)V(t^*)\right]Z \geq 0,$$

which implies that $E(t)$ is non-decreasing at $t = t^*$. Therefore, $E(t)$ cannot be the first component to become negative, leading to a contradiction. Hence, $E(t) \geq 0$ for all $t \geq 0$. Using a similar approach, it can be seen that $I(t), A(t), R(t), D(t), X(t), Y(t)$, and $Z(t)$ also remain non-negative for all $t \geq 0$. Hence, the solution of the initial value problem remains non-negative for all $t \geq 0$.

Next, we need to address boundedness of solutions. We let $N(t)$ be the total human population at time t and $M(t)$ be the total mosquito population at time t. Summing the equations in system S1 (Eq 2) yields $N'(t) = 0$ and hence, the total human population across all compartments remains constant for all $t \geq 0$. Hence, since the total human population is bounded and the components of the solution are non-negative, it follows that each component of the human population remains bounded for all $t \geq 0$. Next, summing the the equations in system S1 (Eq 3) yields the differential equation

$$\frac{\mathrm{d}M}{\mathrm{d}t} = \delta(T,\zeta)Me^{-\sigma M} - \mu(T)M.$$

We need not assume that δ and μ are constants, and we assume only that these parameters are bounded for all t and achieve maximum and minimum values, as is sensible for temperature-dependent parameters. In particular, these functions are continuous on their compact domains of definitions, assuring the existence of global maxima and minima. Let $\tilde{\delta} := \max_{T,\zeta}\delta(T,\zeta)$ and $\tilde{\mu} := \min_T \mu(T)$. Then,

$$\frac{\mathrm{d}M}{\mathrm{d}t} \leq \tilde{\delta}Me^{-\sigma M} - \tilde{\mu}M.$$

Then it follows from a comparison argument that

$$\limsup_{t\to\infty} M(t) \leq \max\left\{0, \frac{1}{\sigma}\ln\left(\frac{\tilde{\delta}}{\tilde{\mu}}\right)\right\}.$$

Therefore, the components of M, which are non-negative, must all remain bounded for all $t \geq 0$. We summarize these results in the following theorem.

Theorem 3.1 *Consider system S1 (Eq 2) – (Eq 3) subject to non-negative initial conditions. Then there exists a unique non-negative solution that remains bounded for all* $t \geq 0$.
