# Supplementary material for: Risk assessment and mitigation evaluation of future yellow fever outbreaks under different climate scenarios: Insight from a case study of Brazil
Source: PLoS Negl Trop Dis. 2025 Oct 16;19(10):e0013448. doi: 10.1371/journal.pntd.0013448 (PMC12543279; doi:10.1371/journal.pntd.0013448)
Supplement: Attachment — Submitted filename: Response to Reviewers.pdf [file pntd.0013448.s005.pdf]

Subject: Response to Reviewers

June 18, 2025

Dear Editor,

Thank you for handling our submission and for securing highly constructive comments from three reviewers. We believe we have addressed their concerns, and our one-to-one response has documented the corresponding revision.

During the revision process, we came to realize that it is the basic human infection reproduction number (rather than the basic reproduction number as defined in the original version), the total number of human infections produced by an infected mosquito, that measures the outbreak potential in the community. Accordingly, we have updated Figures 5 and 6 to reflect this change and highlighted the quantitative differences in blue.

In order to enhance reproducibility, we also re-ran all simulations using consistent initial conditions, parameters, and solver settings across all scenarios. While these revisions led to minor quantitative adjustments, the overall conclusions of the study remain unchanged.

Sincerely,

Tedi Ramaj

Department of Mathematics and Statistics
Laboratory for Industrial and Applied Mathematics
York University
Toronto, ON, Canada

tediramaj95@gmail.com
tramaj@yorku.ca

Dear Reviewer 1,

Thank you for your various remarks and comments. We are glad you found the methodology sound and the results clear. We have addressed your minor comments in the revised manuscript, with the changes highlighted in blue. Please find our more detailed response to the more major comments below.

- ***Comment:*** *One omission from the manuscript as it stands is a hypothesis for why the increase in YF is found here, and does it change the shape of the epidemic curve? E.g., are increasing temperatures increasing vector development rates or is rainfall more related to vector breeding or higher humidity?*

  **Response:** This is a very important question. We have shown simulations that our model gives for different temperature and rainfall values, e.g., see Figure 3. In terms of cumulative infections, we can see a comparison between the 2017/2018 scenario and the RCP8.5 scenario. It can be seen here that while the *shape* of the epidemic curve remains unchanged, there is a significant increase in cumulative cases that accompanies increasing temperatures and rainfall.

  Regarding development rates: Yes, it is absolutely correct that temperature (and rainfall) have an impact on mosquito development and this is reflected in our model through the equations we use to model mosquito development (see Appendix A). This model captures both the effects of temperature and rainfall (and higher humidity) on vector breeding. We have elaborated on this in the methodology section to clarify it.

- ***Comment:*** *Line 84: "We use data from the 2017/2018 YF outbreak in Brazil" - it would be good to know what this data looked like in time and space?*

  **Response:** Please see Figure 2 in the manuscript or the corresponding references [17, 24].

- ***Comment:*** *Line 263: "does not take into account the historical vaccination campaign" surely this is a significant issue here as the vaccine provides immunity for a lifetime.*

  **Response:** In this statement, we simply mean that our model does not account for it in the equations we use. **It is however still accounted for in the sense of how many individuals are initially assumed to have immunity in the model.** Our model then looks at the impact of additional/emergency vaccines distributed to portions of the population that are not yet vaccinated. Thank you for pointing out the lack of clarity in this statement. We have updated the manuscript to make the meaning more clear.

The remaining minor comments have all been addressed in the main text, highlighted in blue. The authors feel that these changes have made the manuscript much stronger and we once again extend our gratitude to the reviewer.

Dear Reviewer 2,

We would like to thank you for your time and effort in reviewing our manuscript. We have found your comments to be very insightful in improving the quality of our manuscript and we greatly appreciate all of the helpful suggestions. The changes, both in the main body of the manuscript and in the bibliography, may be found highlighted in blue. Below, we have also provided detailed responses addressing your comments and remarks.

- ***Comment:*** *One point I would consider is if there are any examples of non-modeled increases in YF (or any other mosquito born pathogen) prevalence or impact due to climate change (i.e., actual data from South America) you can reference? For example, have case loads increased across the last 20 years or change their distributions to more temperate areas? Added in the references could help really nail down why this study is so important.*

  **Response**: Thank you for the helpful suggestion. We agree that including real-world evidence of changes in Yellow Fever (YF) transmission strengthens the case for our study. We have observed recent YF outbreaks in regions previously considered low risk, such as São Paulo and Minas Gerais in Brazil, as well as West and Central Africa in 2021 (these are now mentioned in the paper). These events suggest that environmental changes including climate warming are facilitating shifts in disease distribution. We also note the relevance of other arboviruses such as dengue, Zika, and chikungunya, which have increased significantly in recent decades and expanded into new regions, including local transmissions in North America and a few in Europe. For example, local dengue outbreaks in Madeira, Portugal, were linked to the establishment of Aedes aegypti in the early 2000s (Schaffner & Mathis, 2014), illustrating how changing environmental conditions have facilitated the spread of competent vectors. The expansion of other arboviruses into new regions illustrates how similar environmental changes could facilitate the spread of Yellow Fever as well.

- ***Comment:*** *I would also consider taking some space to explain what type or epidemiological model you are using and why it is appropriate in your introduction*

  **Response**: The type of model we are using, which has seen widespread and successful use in epidemiological modeling is a compartmental SEAIR (Susceptible-Exposed-Asymptomatic Infectious-Symptomatic Infectious-Recovered) differential equation model. This is a commonly used type of differential equation model in the mathematical modelling literature We have discussed this in our methodology section but we agree that it is a good idea to also mention this in the introduction section as you have suggested. We have made this change as per your suggestion. (See the bottom of page 3.)

- ***Comment:*** *You could also use this space to highlight your incorporation of vaccination coverage in your model, and why that is both novel and important. Specifically, some points that you make in your discussion about vaccination coverage in this region prior to the outbreak could be moved here to give the reader a bit more context on this specific outbreak.*

  **Response:** Excellent point. It has been done.

- ***Comment:*** *Page 2 line 16- Define WHO and EYE acronyms.*

  **Response:** Thank you for this comment. We have done so on page 2, lines 17 and 18.

- ***Comment:*** *Lines 28-29: Sentence "Certain regions currently endemic to YF. . . ." Needs a citation.*

  **Response:** Reference added on page 3, line 29.

- ***Comment:*** *you state that parameters v and n are either assumed or derived but you do not prompt the reader to go to Table 2 until the end of this section (i.e., lines 65-86). It is then unclear where the parameters were assumed or calculated from if you are just looking at the table. I would suggest clarifying early on in this section that all parameters were either derived from the case study outbreak (and cite where you got that data from), or were based on previously published literature on this disease system. Again, the details are there, they were just a little difficult to find at times.*

  **Response:** Thank you for this suggestion. This has been done by clarifying early on that all parameters were either derived from the case study or based on previously established literature.

- ***Comment****: In that same vein, move the sentence from lines 131-134 (source of temp and rainfall projections) up to your methods.*

  **Response:** We have modified the model section and included the source in that chapter.

- ***Comment****: Consider moving Fig 2 down to results and also reporting the performance of the fitted model. The figure is informative, but putting some numbers with it would send home the message that this model is well calibrated to the data.*

  **Response:** We have kept this figure in the methodology section since this is not technically a result of our model. We are just showing the fit which resulted from our calibration. As we are not using a linear model, we report the Root Mean Square Error (RMSE) instead of the $R^2$ value. The RMSE is approximately 31.6 infections, primarily due to the model underestimating the outbreak peak. Nevertheless, the model accurately captures the overall shape, timing, and qualitative behavior of the epidemic curve, supporting its use for evaluating intervention strategies.

- ***Comment****: Inversely, sentences from lines 106-107 and 118-119 about how you simulated the outbreak under different temp and rainfall values may belong in your methods section.*

  **Response:** This is a great point. We have included the lines about simulating the outbreak under different rainfall and temperature values in our methods section. See the blue highlighted text on page 7.

- ***Comment****: One question that I have regarding your projections: Are the average temperatures and rainfall values that you are using averaged across the year or are they the average values for a specific season? You report increases in cases based by week of the outbreak, but if I am not mistaken, the temp and rainfall numbers for each scenario are static and used to calculate the mosquito metrics. Do I have that correct, or am I misreading? This was unclear to a certain extent. As a follow up to that, does YF have seasonal outbreak patterns in this region? How might climate change impact the seasonality of temp and rainfall in this region, and might that impact YF outbreaks? Assessing seasonality might be beyond the scope of this study, but it may be worth noting in your discussion.*

**Response:** Thank you for bringing up this excellent point. Yes, we have used **average** temperature and rainfall values. By this, we mean that we take the average temperature and rainfall values in the region over the course of the historical outbreak (i.e., by averaging weekly values). This serves many purposes, such as mathematical and computational tractability of the model. We have now clarified this use of average temperature and rainfall values in the methodology.

While you are correct that assessing seasonality is beyond the scope of this particular manuscript, this could certainly be an important topic to consider for future research. As per the reviewer's suggestion, we have put this in the discussion.

- ***Comment:*** *Figure 9: What is the slope of the line for cumulative infections?*

  **Response**: While the relationship may appear linear, it is actually nonlinear - it is a curve generated by running the compartmental model and calculating the cumulative infections as the mosquito-to-human transmission rate varies.

- ***Comment:*** *Was there a specific software that you used to run your model? If so, specify it and any packages you used in your methods section.*

  **Response**: Yes, we used Python to run the calibrate the model and run the simulations, primarily using packages like **numpy** and **scipy**. We have also added a paragraph in the methods section specifying this.

- ***Comment:*** *Lines 226-231: This is a great detail that may be worth moving up to the introduction and/or being expanded in the discussion section. What are some challenges to emergency vaccination in this region? This could help tie in how your results are useful for public health officials.*

  **Response**: Thank you for this thoughtful comment. Yes we agree and have expanded this discussion, along with an appropriate citation. We believe this further clarifies the importance of emergency vaccination strategies in high-risk areas. Please see the blue highlighted text starting on line 243.

- ***Comment:*** *Lines 283-284: Sentence, "Such countries typically have highly susceptible populations" needs a citation.*

  **Response**: We have reformulated this line in a way that does not make such a statement, while still making our point clear. Please see the revised line in highlighted blue text in line 304.

- ***Comment****: Is there a particular reason why you chose to use RCP's over more contemporary SSPs in CMIP 6 for your future climate projections? If so, introduce this in your methods or even introduction.*

  **Response:** One of our main motivations for using RCPs over SSPs is the mathematical and computational tractability. In particular, developing models and running simulations can be more feasible since they provide us with direct means of quantifying temperature and rainfall changes over time. SSPs on the other hand are based more on human behaviour. While this is certainly a very important aspect to study, it falls beyond the scope of this kind of modeling.

- ***Comment**: Make sure all of your methods are in the methods section and that all your results are in the results section. I found myself having questions about how you did some assessments (particularly the vaccine assessment) after reading your methods section, only to have those questions answered later on in the results. This just helps me as the reader have the full story before I move on into your results and discussion.*

  **Response:** Thank you for this comment. Yes, we agree that this is an important consideration and have made the changes accordingly. In particular, more details concerning vaccine assessment were added into the methods section.

The remaining minor comments have been addressed in the main text, highlighted in blue. The authors once again extend our gratitude to the reviewer for the detailed and constructive review which has greatly improved the quality of the manuscript.

Dear Reviewer 3,

Thank you for taking the time to carefully read and review our manuscript. We appreciate the time you took to verify that you found the presentation of the results to be clear and the methodology used to be sound.
